# Cryptorchidism and Maternal Alcohol Consumption during Pregnancy

**DOI:** 10.1289/ehp.9608

**Published:** 2006-12-04

**Authors:** Ida N. Damgaard, Tina K. Jensen, Jørgen H. Petersen, Niels E. Skakkebæk, Jorma Toppari, Katharina M. Main, the Nordic Cryptorchidism Study Group

**Affiliations:** 1 University Department of Growth and Reproduction, Rigshospitalet, Copenhagen, Denmark; 2 Departments of Physiology and Pediatrics, University of Turku, Turku, Finland; 3 Department of Biostatistics, University of Copenhagen, Copenhagen, Denmark

**Keywords:** alcohol, caffeine, cohort studies, cryptorchidism, risk factors, smoking

## Abstract

**Background:**

Prenatal exposure to alcohol can adversely affect the fetus. We investigated the association between maternal alcohol consumption during pregnancy and cryptorchidism (undescended testis) among newborn boys.

**Methods:**

We examined 2,496 boys in a prospective Danish–Finnish birth cohort study for cryptorchidism at birth (cryptorchid/healthy: 128/2,368) and at 3 months of age (33/2,215). Quantitative information on alcohol consumption (average weekly consumption of wine, beer, and spirits and number of binge episodes), smoking, and caffeine intake was obtained by questionnaire and/or interview once during the third trimester of pregnancy, before the outcome of the pregnancy was known. For a subgroup (*n* = 465), information on alcohol consumption was obtained twice during pregnancy by interviews.

**Results:**

We investigated maternal alcohol consumption both as a continuous variable and categorized. The odds for cryptorchidism increased with increasing weekly alcohol consumption. After adjustment for confounders (country, smoking, caffeine intake, binge episodes, social class, maternal age, parity, maturity, and birth weight) the odds remained significant for women with a weekly consumption of five or more alcoholic drinks (odds ratio = 3.10; 95% confidence interval, 1.05–9.10).

**Conclusions:**

Regular alcohol intake during pregnancy appears to increase the risk of congenital cryptorchidism in boys. The mechanisms for this association are unknown. Counseling of pregnant women with regard to alcohol consumption should also consider this new finding.

Although cryptorchidism is one of the most common genital malformations in males, its etiology remains largely unknown. Defects in developmental genes such as *INSL3* (insulin-like factor 3) seem to be involved in some cases (Adham and Agoulnik 2004). Other risk factors include low birth weight, prematurity, low parity, and twinning (Hjertkvist et al. 1989). Several studies indicate an increase in the prevalence of cryptorchidism within a few generations, supporting the hypothesis that lifestyle changes and environmental factors may be involved (Berkowitz et al. 1993; Boisen et al. 2004; John Radcliffe Hospital Cryptorchidism Study Group 1992; Paulozzi 1999).

Normal testicular descent occurs in two phases: the first phase (transabdominal) between gestational weeks 8 and 17 and the second phase (inguino–scrotal) between weeks 26 and 35 (Toppari and Kaleva 1999). Thus, testicular descent may be vulnerable to adverse lifestyle and environmental factors throughout pregnancy.

Maternal alcohol consumption during pregnancy has been associated with adverse outcomes such as low birth weight, miscarriage, stillbirth, and fetal alcohol syndrome (FAS) (Armstrong et al. 1992; Clarren and Smith 1978; Henriksen et al. 2004; Kesmodel et al. 2002; Windham et al. 1995). Children with FAS have more skeletal anomalies, cardiac defects, and genitourinary malformations than the general population (Clarren and Smith 1978; Hadi et al. 1987). Other lifestyle factors associated with alcohol consumption, such as smoking and caffeine intake, have also been associated with low birth weight, stillbirth, and miscarriage (Armstrong et al. 1992; Cnattingius et al. 2000; Peacock et al. 1991; Wisborg et al. 2001, 2003).

A few case–control studies of cryptorchidism did not find any significant associations between maternal alcohol consumption and cryptorchidism (Berkowitz and Lapinski 1996; Biggs et al. 2002; Davies et al. 1986; Kurahashi et al. 2005; McBride et al. 1991; Møller and Skakkebæk 1997). In general, these studies included retrospective data without detailed information on alcohol intake and may be compromised by recall and selection bias. We assessed the relationship between maternal alcohol consumption during pregnancy and congenital cryptorchidism in a prospective, population-based cohort of pregnant women and their male offspring. We obtained qualitative and quantitative information on alcohol consumption during pregnancy together with data on other confounding lifestyle factors.

## Materials and Methods

We performed a joint prospective birth cohort study at the University Hospital of Copenhagen (Rigshospitalet and Hvidovre Hospital) in Denmark from 1997 to 2001 and at the Turku University Central Hospital in Finland in the period 1997–1999. The cohort (antenatal recruitment, inclusion criteria and clinical examinations) has previously been described in detail (Boisen et al. 2004). Although the study was conducted in two different countries, the design, questionnaires, and examinations were strictly standardized. We previously reported that the prevalence of cryptorchidism was higher in Denmark than in Finland, which was primarily due to transient and mild forms of cryptorchidism (Boisen et al. 2004).

**Figure 1:**
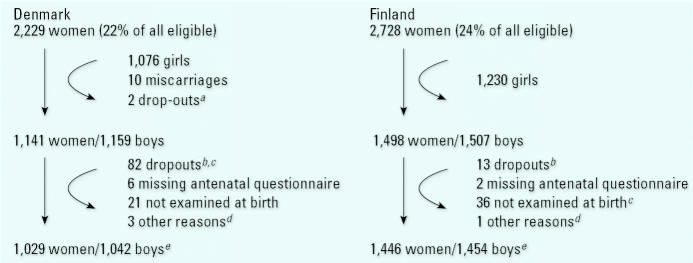
Flow chart of the total number of women included in early pregnancy in a joint Danish–Finnish birth cohort study, and numbers of dropouts during follow-up before and after birth. ^***a***^Sex of child unknown. ^***b***^Denmark: 4 moved, 44 uninterested, 3 child deaths, 7 child sickness, 17 lost, 7 other reasons; Finland: 13 lost. ^***c***^Denmark: including 5 pairs of male–male twins; Finland: including 1 pair of male–male twins. ^***d***^Denmark: 1 unilateral torsion, 1 could not be classified for the position of the testes at birth due to severe bilateral inguinal hernia, and 1 not classified at birth; Finland: 1 unilateral agenesis testis. ^***e***^Denmark: 45 twins including 13 pairs of male–male twins; Finland: 20 twins including 8 pairs of male–male twins.

Eligible women (2,229 Danish and 2,728 Finnish) residing in the hospital referral areas were consecutively recruited during early pregnancy, by mail (Denmark) or at the first antenatal visit (Finland). Women referred from outside the recruitment area because of pregnancy complications were not included. To obtain well-defined populations, only families who met the following criteria were included: Both parents and grandparents of the unborn child had to be born and raised in Denmark or Finland. A maximum residence abroad of 3 years for the mother and 10 years for the father and grandparents was allowed. The total numbers of included families and numbers lost for follow-up are shown in Figure 1.

After informed written consent, the women received a questionnaire by mail late in the first or early in the second trimester, covering medical and obstetric history, lifestyle, education, working conditions, and family history of urogenital malformations. We instructed the women to complete the questionnaire at the beginning of the third trimester and to return it by mail before birth.

The following question was asked: “How much have you on average been drinking during this pregnancy?” Response categories were the average number of glasses of wine per week, bottles of beer per week, liquor glasses of spirits per week. One alcoholic drink was defined as one glass of wine, one bottle of beer (0.33 L), or one liqueur glass of spirits. These were added up to a total. The women were also asked about the number of binge episodes—episodes where they had been noticeably inebriated (never, 1–2 times, ≥ 3 times). Average daily consumption of tea and coffee in cups per day was also registered. Caffeine intake from tea and coffee was grouped into 0–100, 100–300, and > 300 mg caffeine per day. One cup of coffee was set to contain on average 115 mg of caffeine and one cup of tea 39 mg (Cnattingius et al. 2000).

Data on smoking, including changes in smoking habits during pregnancy, were also obtained. Smoking at any time during pregnancy was categorized as “smokers,” whereas women who had never smoked were categorized as “nonsmokers.” Social class was determined from occupational status of the mother in seven hierarchical categories: higher-grade professionals, lower-grade professionals, skilled workers, unskilled workers, students, economically inactive, and unclassifiable (Albertsen et al. 2004). Unclassifiable was treated as missing values.

Simultaneous with our cohort but independent of it, a national birth cohort study was conducted [The Danish National Birth Cohort (DNBC)] in Denmark (Olsen et al. 2001). Women participating in this study completed two telephone interviews during pregnancy, which contained most of the questions asked in our self-administered questionnaire. Information on alcohol consumption was collected in both interviews. In total, 495 Danish women from our study were also included in the DNBC and participated in the two interviews. Of these, a subgroup of 210 women completed a shortened questionnaire, which supplemented information lacking in the interviews. Two hundred eighty-five women completed the entire questionnaire to assess potential differences between answers obtained by questionnaire and telephone interviews. The interviews of the national study and our questionnaires were developed in close collaboration and were therefore almost identical. In the telephone interview, the corresponding questions were “How many glasses of wine and bottles of beer and glasses of spirits, respectively, do you drink per week?” and “How many cups of tea and coffee, respectively, do you drink per week?” *A priori*, the responses were categorized as none, less than one per week, the precise number of drinks per week, or do not know/do not wish to answer. The women were also asked about binge episodes—number of episodes drinking five or more alcoholic drinks at one occasion/day (none, the accurate number of times, do not know/do not wish to answer). The response category “do not know/do not wish to answer” was treated as missing values in the analysis, and the category “< 1 drink/week” was analyzed as 0.5 drink/week.

The study was conducted according to the Helsinki II Declaration (World Medical Association 2004) and was approved by the local ethics committees in both countries (Finland: 7/1996; Denmark: KF01-030/97) and the Danish Data Protection Agency (registration no. 1997-1200-074, 2001-3311-0068).

### Clinical examination

All boys were examined at birth and at 3 months of age. Preterm boys were examined at the expected date of delivery. Gestational age was based on routine ultrasonography performed in pregnancy weeks 18–20. If not available, the last menstrual period was used (2.1%). Testicular position was described after manipulation of the testis to the most distal position along the pathway of normal descent, using firm but not forced traction. The testis was considered cryptorchid if found in a high scrotal, supra-scrotal, inguinal position or was nonpalpable. Retractile testis was considered to be a normal variant (Boisen et al. 2004). To minimize interobserver variation, borderline cases were examined by two researchers, and binational workshops were held regularly.

The results presented in Table 1–4 are based on the diagnosis at the newborn examination and the 3 months examination, respectively, without dividing into cryptorchidism subtypes. Information on birth weight and parity was collected from hospital records.

## Statistical Analysis

Descriptive data are given as numbers and percentages. Gestational age for completing questionnaires and interviews is given as mean ± SD and differences were tested by unpaired *t*-tests.

We studied maternal alcohol intake during pregnancy both as a continuous variable (number of alcoholic drinks/week) and categorized into groups. Differences in maternal alcohol consumption between mothers of cryptorchid and healthy boys were tested by chi-square test (two-sided) or Fisher’s exact test (two-sided) or described by odds ratios (ORs) and 95% confidence intervals (CIs). We estimated ORs (unadjusted and adjusted) by logistic regression analysis. Due to a known country difference in the birth prevalence of cryptorchidism, we always adjusted for country of origin. Analyses including other potential confounders (smoking, caffeine intake, binge episodes, social class, maternal age, parity, maturity, and birth weight) were also performed. Binge drinkers without a regular alcohol intake were included in the “0 alcoholic drinks/week” category, although they were not total abstainers.

In the group of women with no regular weekly alcohol consumption (*n* = 1,719), 439 reported one or more episodes of binge drinking. To account for the possibility that total abstainers may constitute a selected group, we included also an indicator variable (abstainer/nonabstainer) when analyzing alcohol consumption as a continuous variable. In this way the linear alcohol effect was assumed only for those who did consume alcohol, and the effect of being abstainer was estimated separately.

Answers to questions on alcohol consumption and binge episodes were missing more frequently in mothers of cryptorchid than in mothers of healthy boys [2 (1.6%) vs. 17 (0.7%), *p* = 0.254; and 7 (5.5%) vs. 45 (1.9%), *p* = 0.015, respectively]. Missing data were categorized as 0 alcoholic drinks/week and no binge episodes, respectively, in a sensitivity analysis. We validated the comparability of answers (percentage of agreement) among women (*n* = 285) who completed both the questionnaire and the second interview.

## Results

In Denmark, 1,042 boys (1,029 mothers) and in Finland 1,454 boys (1,446 mothers) participated in the study (Figure 1). In total, 128 boys (94 Danish, 34 Finnish) were cryptorchid at birth, and 33 (19 Danish, 14 Finnish) remained cryptorchid at 3 months of age.

Most questionnaires (94%) were returned before birth, but in 104 women the return date had not been registered [12 (9.4%) mothers of cryptorchid boys and 92 (3.9%) mothers of normal boys, *p* = 0.002]. Two mothers of cryptorchid boys and 37 mothers of normal boys completed the questionnaire after birth (*p* = 0.687). Mean gestational age for completion of the questionnaire/second interview for mothers of cryptorchid boys was 199 days ± 41 versus 207 days ± 58 for mothers of normal boys (*p* = 0.211). The corresponding figures for the first interview were 113 ± 33 days and 115 ± 30 days (*p* = 0.755).

Table 1 shows the distribution of maternal alcohol consumption during pregnancy among normal and cryptorchid boys. In total, 758 (30.6%) mothers reported a weekly intake of alcohol (19 missing), and 736 (30.1%) had experienced one or more binge episodes (52 missing). More mothers of cryptorchid boys (*n* = 55, 43.7%) than those of normal boys (*n* = 703, 29.9%) reported a weekly intake of alcohol (*p* < 0.001); 17 mothers of cryptorchid boys (14%) and 422 mothers of normal boys (18%) had experienced one or more binge episodes during pregnancy without regular weekly consumption of alcohol, *p* = 0.249.

Compared with mothers with an average consumption of less than five alcoholic drinks/week, mothers consuming five or more alcoholic drinks/week were older, they had more children, their daily intake of caffeine was higher, but there was no difference in social class (Table 2). Binge episodes were more common among them (64.7% vs. 29.6%, *p* < 0.001). Their children were more frequently cryptorchid than those of women who consumed less alcohol (17.6% vs. 4.9% (*p* = 0.006), whereas birth weight < 2,500 g was statistically as frequent in both groups (5.9% vs. 2.6%, *p* = 0.229). More Danish than Finnish women had a weekly consumption of five or more alcoholic drinks (64.7% vs. 35.3%, *p* = 0.005).

Maternal alcohol consumption during pregnancy was associated with an increased risk of cryptorchidism (adjusted OR = 1.17; 95% CI, 1.03–1.34) (Table 3). Comparable results were obtained in analyses including an indicator variable for total abstainers (adjusted OR = 1.22; 95% CI, 1.06–1.39). For grouped alcohol data the adjusted odds ratio was significant for women consuming five or more alcoholic drinks/week (OR = 3.10; 95% CI, 1.05–9.10). Including only boys with a birth weight ≥ 2,500 g strengthened the results (adjusted OR = 3.72; 95% CI, 1.23–11.16). Excluding twins (*n* = 65) did not change the findings (adjusted OR = 3.53, 95% CI, 1.20–10.42). Analysis of both countries separately showed comparable results, which, however, did not reach statistical significance due to reduced sample size (data not shown).

Binge episodes increased the OR for cryptorchidism (unadjusted OR = 1.25; 95% CI, 0.85–1.87; adjusted OR = 1.18, 95% CI, 0.77–1.83), but not significantly. Stratified analyses of alcohol consumption during pregnancy (yes/no) and binge episodes (yes/no) revealed that regular alcohol consumption was the main determinant for the risk of cryptorchidism.

Including missing data concerning weekly alcohol consumption and binge episodes as 0 drinks/week and no binge episodes, respectively, in the analyses did not substantially change the estimates (≥ 5 drinks/week vs. 0 drinks/week: adjusted OR = 3.22; 95% CI, 1.10–9.46; binge drinking: adjusted OR = 1.18; 95% CI, 0.81–1.72). Excluding women with an unknown date of questionnaire return or postnatal return did not change the results (≥ 5 drinks/week vs. 0 drinks/week adjusted OR = 3.28; 95% CI, 1.10–9.76).

In total, 33 boys remained cryptorchid at 3 months of age (persistent cryptorchidism). The overall drinking pattern of their mothers resembled that of mothers of normal boys (Table 4). Due to sample size, no further sub-analyses were performed.

Of 465 women who participated in both interviews, 89.7% reported unchanged alcohol consumption (± 1 drink/week), 1.7% a lower, and 8.6% a higher consumption in the second interview. There was a high comparability between answers given in the questionnaire and the interview. An agreement of ± 1 drink/week was found in 95, 92, and 99% for wine, beer, and spirits, respectively.

## Discussion

We found a significantly increased risk for congenital cryptorchidism if mothers reported a regular intake of alcohol during pregnancy. In this study we focused on boys with congenital cryptorchidism. Although many of these were mild and transient forms of cryptorchidism, in which testes descend spontaneously without treatment (Boisen et al. 2004), these boys show slightly elevated gonadotropin levels at 3 months of age as an indication of subtle primary testis dysfunction (Suomi et al. 2006). The number of boys with persistent cryptorchidism was too small to make separate analyses. However, because Danish women had a higher alcohol intake than Finnish women, and mild and transient cryptorchidism was more frequent in Denmark than in Finland (Boisen et al. 2004), our findings suggest that maternal alcohol consumption contributes to the geographical difference in the prevalence of cryptorchidism and the observed increase in the Danish population.

Only a few mothers had a high weekly alcohol consumption, and we therefore conducted the analyses with alcohol intake as a continuous variable and grouped. We found that the odds for cryptorchidism increased with weekly alcohol consumption and became statistically significant when consumption increased to five or more alcoholic drinks per week. However, because the OR increased linearly, our data should not be interpreted toward a definite threshold of five alcoholic drinks per week. The observed association may well be a continuous phenomenon.

There is as yet no well-established safety level of drinking during pregnancy. In 1999, the Danish National Board of Health adjusted the recommendations for pregnant women in Denmark from the previously recommended “total abstain” to: “avoid drinking if possible, and if drinking then only 1 alcoholic drink/day and not every day” (Sundhedsstyrelsen 1999). After having reviewed the literature, regular intake of approximately one alcoholic drink per day was considered potentially harmful with respect to other fetal outcomes such as low birth weight and miscarriages (Sundhedsstyrelsen 1999). These data correspond well to our findings that five or more alcoholic drinks per week appeared to affect testicular descent.

Most previous studies of cryptorchidism and maternal alcohol consumption have found no associations. In general, these studies had no detailed information on maternal alcohol consumption and often included retrospective data obtained after the outcome of the pregnancy was known (Berkowitz and Lapinski 1996; Biggs et al. 2002; Davies et al. 1986; Kurahashi et al. 2005; McBride et al. 1991; Møller and Skakkebæk 1997). Thus, these studies may be influenced by recall and selection bias. Finally, many of the studies have been based on registry data for cryptorchidism, which may be limited by variations in ascertainment of the diagnosis and reporting strategies (Toppari et al. 2001).

Animal studies have provided evidence that binge drinking may be more harmful than small amounts of alcohol over a long time (Bonthius and West 1990). However, human studies are more inconsistent [for references, see Kesmodel (2001)]. In our study there was little relationship between binge drinking and the risk of cryptorchidism, whether or not there was a regular alcohol consumption.

Genitourinary malformations have been described as occurring more commonly in children with FAS than in general (Clarren and Smith 1978; Havers et al. 1980; Qazi et al. 1979). A few animal studies have found that fetal alcohol exposure may result in abnormalities of the genitourinary tract (Gage and Sulik 1991; Randall et al. 1977). Others have described reduced anogenital distance, reduced growth of the testes, and delayed attainment of adult levels of testosterone in male rats exposed to ethanol *in utero* (Udani et al. 1985). In humans, serum maternal testosterone was decreased by alcohol consumption (Stevens et al. 2005).

The adverse effect of alcohol may depend on or be confounded by simultaneous exposure to other factors such as smoking and caffeine (Armstrong et al. 1992; Peacock et al. 1991; Sundhedsstyrelsen 1999; Windham et al. 1995). In our study, mothers with a high alcohol consumption also were more frequently smokers and had a higher caffeine intake. Although these factors alone did not significantly increase the risk of cryptorchidism (data not shown), they may still contribute to an overall adverse effect. Socioeconomic status is related to drinking habits and in some studies also to the risk of cryptorchidism (Czeizel et al. 1981; Møller and Skakkebæk 1996). However, in our study the social class of the mother consuming five or more alcoholic drinks/week did not differ significantly from mothers reporting lower consumption. The prevalence of drinking in our cohort may not be representative of the entire Danish and Finnish population, because only 22–24% of all eligible women participated in our study, and mothers with an academic degree were overrepresented. However, this should not bias our estimates of the association between alcohol and cryptorchidism, because the data were collected prospectively and therefore any misclassification of alcohol exposure such as underreporting is most likely to be nondifferential, causing a bias toward the null hypothesis. Furthermore, the prospective design minimizes the risk of selection bias among normal boys. The reported alcohol consumption was in line with previous data from two national Danish birth cohorts born in 1998 and 1999, in which 6.4% and 7.4%, respectively, of the pregnant women reported to consume more than two alcoholic drinks per week, and 26.4 and 24.7%, respectively, described binge episodes (Andersen et al. 2001).

There was good agreement between the information obtained by the interview and the questionnaire, and we would therefore not expect any bias from this difference in study design. However, we cannot exclude the possibility that some information bias may have been introduced by the structure of the questionnaire and the interview, because some mothers were included in the 0 alcoholic drinks/week category, although they were not total abstainers. This classification may have diminished the risk of cryptorchidism observed between mothers with a regular alcohol intake and mothers without a regular intake.

We did not systematically obtain information about changes in alcohol consumption during pregnancy for all participants and could therefore not differentiate the amount of alcohol consumption within each trimester. Women may reduce their alcohol consumption after discovering that they are pregnant (Fried et al. 1980; Little 1982; Sulaiman et al. 1988) One study found a constant consumption through the remaining part of the pregnancy, whereas another described a further decrease (Little 1982; Sulaiman et al. 1988). One study indicated that alcohol consumption may be relatively constant for small amounts, whereas there may be a decrease for women with the highest consumption (Fried et al. 1980). In our study, the women who participated in both interviews did not substantially change their alcohol consumption during pregnancy.

We included twins in the analyses, although their observations are not independent. This did not significantly influence the results because the number of twins was low (Figure 1). Maternal alcohol consumption during pregnancy may result in low birth weight, which in itself is a risk factor for cryptorchidism (Boisen et al. 2004). However, the difference caused by alcohol remained statistically significant after controlling for birth weight or the inclusion of only boys with a normal birth weight in the analyses.

Although maternal alcohol consumption during pregnancy has been associated with adverse outcomes such as FAS, stillbirth, miscarriage, being small-for-gestational age, low birth weight, pre- and postmature birth, and abruptio placentae, the precise mechanisms are not known. Two main mechanisms have been proposed (Hadi et al. 1987). Ethanol or its metabolites may directly affect cellular functions or interfere with absorption, transportation, and utilization of other substances. Ethanol has a high lipid solubility and can cross the placenta. The levels detected in the embryo are similar to those of the mother. The adverse effects seem to depend not only on ethanol concentrations but also on the duration and timing during embryogenesis (Bonthius and West 1990). In addition, genetic susceptibility to the adverse effect of alcohol can vary between strains of mice after the same degree of exposure (Ogawa et al. 2005).

In conclusion, we found an increased risk of congenital cryptorchidism among boys born of mothers with a regular alcohol consumption during pregnancy compared with mothers who abstained from alcohol. This suggests that maternal lifestyle plays a role in testicular descent in humans. The mechanims responsible for this adverse effect of alcohol are unknown, but the findings warrant further studies. Counseling of pregnant women with regard to alcohol consumption should consider this new finding.

## Figures and Tables

**Table 1:** Self-reported maternal alcohol consumption during pregnancy in relation to cryptorchidism among the offspring in a joint Danish–Finnish birth cohort study conducted 1997–2001 [no. (%)].

	Cryptorchid boys (*n* = 128)	Normal boys (*n* = 2,368)	*p*-Value
Reported average consumption of alcohol during pregnancy (alcoholic drinks/week)			
0	71 (56.3)	1,648 (70.1)	
0.1–0.9	12 (9.5)	167 (7.1)	
1.0–1.9	17 (13.5)	288 (12.3)	
2.0–2.9	15 (11.9)	133 (5.7)	< 0.001
3.0–4.9	5 (4.0)	87 (3.7)	
≥ 5	6 (4.8)	28 (1.2)	
Binge episodes during pregnancy			
0	79 (65.3)	1,629 (70.1)	
1–2	38 (31.4)	627 (27.0)	0.528
≥ 3	4 (3.3)	67 (2.9)	
Types of beverages^a^			
Glasses of wine/week			
0	71 (67.0)	1,648 (79.7)	0.002
> 0	35 (33.0)	419 (20.3)	
Bottles of beer/week			
0	71 (95.9)	1,648 (92.8)	0.300
> 0	3 (4.1)	128 (7.2)	
Liquor glasses of spirits/week			
0	71 (100.0)	1,648 (99.7)	1.000
> 0	0 (0)	5 (0.3)	

The *p-*value (two-sided) describes the difference between cryptorchid and normal boys.

a Comparing women consuming only one type of alcohol with women reporting 0 alcoholic drinks/week.

**Table 2:** Population characteristics and lifestyle factors for mothers consuming < 5 or ≥ 5 alcoholic drinks/week, in a joint Danish–Finnish birth cohort study [no. (%)].

Characteristic	< 5 drinks/week (*n* = 2,443)	≥ 5 drinks/week (*n* = 34)	*p*-Value
Cryptorchidism	120 (4.9)	6 (17.6)	0.006
Country			
Denmark	1,002 (41.0)	22 (64.7)	0.005
Finland	1,441 (59.0)	12 (35.3)	
Maturity (weeks)			
Premature (< 37)	118 (4.8)	0	0.066
Mature (37–42)	2,214 (90.6)	30 (88.2)	
Postmature (> 42)	111 (4.5)	4 (11.8)	
Birth weight (g)			
< 2,500	64 (2.6)	2 (5.9)	
2,500–3,500	884 (36.2)	12 (35.3)	0.502
> 3,500	1,495 (61.2)	20 (58.8)	
Parity			
1	1,424 (58.3)	11 (32.4)	0.002
≥ 2	1,019 (41.7)	23 (67.6)	
Maternal age (years)			
< 30	1,280 (52.4)	4 (11.8)	< 0.001
≥ 30	1,163 (47.6)	30 (88.2)	
Smoking			
Yes	708 (29.0)	15 (44.1)	0.054
No	1,734 (72.0)	19 (55.4)	
Caffeine intake (tea and coffee) (mg/day)			
< 100	926 (37.9)	7 (20.6)	
100–300	969 (39.7)	12 (35.3)	0.008
> 300	548 (22.4)	15 (44.1)	
Social class			
1 + 2: higher- and low-grade professionals	839 (36.9)	12 (40.0)	
3 + 4: skilled and unskilled workers	1,106 (48.4)	13 (43.3)	0.853
5 + 6: students and economically inactive	334 (14.7)	5 (16.7)	
Binge episodes			
Yes	713 (29.6)	22 (64.7)	< 0.001
No	1,696 (70.4)	12 (35.3)	

The *p*-value (two-sided) describes differences between mothers with a low versus high weekly alcohol consumption, respectively.

**Table 3:** Unadjusted and adjusted ORs (95% CIs) for cryptorchidism within categories of maternal alcohol consumption during pregnancy.

	Study population (no.)	OR unadjusted (95% CI)	OR adjusted (95% CI)^a^
Drinks/week continuous	2,477	1.26 (1.13–1.40)	1.17 (1.03–1.34)
Drinks/week			
0	1,719	1 (referent)	1 (referent)
0.1–1	179	1.67 (0.89–3.14)	1.30 (0.65–2.59)
≥ 1	579	1.86 (1.26–2.75)	0.94 (0.58–1.51)
Drinks/week			
0	1,719	1 (referent)	1 (referent)
0.1–1.9	484	1.48 (0.95–2.31)	0.88 (0.53–1.47)
≥ 2	274	2.43 (1.52–3.89)	1.28 (0.72–2.27)
Drinks/week			
0	1,719	1 (referent)	1 (referent)
0.1–2.9	632	1.74 (1.18–2.56)	0.98 (0.62–1.55)
≥ 3	126	2.20 (1.15–4.31)	1.21 (0.55–2.66)
Drinks/week			
0	1,719	1 (referent)	1 (referent)
0.1–3.9	696	1.68 (1.15–2.46)	0.97 (0.62–1.51)
≥ 4	62	3.44 (1.58–7.50)	1.77 (0.67–4.69)
Drinks/week			
0	1,719	1 (referent)	1 (referent)
0.1–4.9	724	1.69 (1.16–2.45)	0.95 (0.61–1.49)
≥ 5	34	4.97 (2.00–12.40)	3.10 (1.05–9.10)
Drinks/week			
0	1,719	1 (referent)	1 (referent)
0.1–5.9	738	1.69 (1.16–2.45)	0.94 (0.61–1.48)
≥ 6	20	7.74 (2.74–21.88)	5.47 (1.59–18.88)
Drinks/week			
0	1,719	1 (referent)	1 (referent)
0.1–6.9	745	1.74 (1.21–2.52)	0.96 (0.61–1.49)
≥ 7	13	6.96 (1.88–25.86)	6.54 (1.56–27.43)
Drinks/week			
0	1,719	1 (referent)	1 (referent)
0.1–7.9	749	1.73 (1.20–2.50)	0.94 (0.60–1.47)
≥ 8	9	11.61 (2.84–47.35)	16.78 (3.48–81.02)
Drinks/week			
0	1,719	1 (referent)	1 (referent)
0.1–8.9	753	1.76 (1.22–2.54)	0.96 (0.62–1.49)
≥ 9	5	15.47 (2.55–94.07)	31.89 (3.96–256.93)
Binge episodes			
Yes	736	1.25 (0.85–1.87)	1.18 (0.77–1.83)
No	1,708	1 (referent)	1 (referent)

Differences between cases and controls were tested by logistic regression.

a Adjusted for country, smoking, caffeine intake, maternal age, social class, parity, maturity, birth weight, and binge episodes and alcoholic drinks/week mutually.

**Table 4:** Distribution of maternal alcohol consumption during pregnancy for mothers giving birth to healthy boys at both birth and 3 months of age, boys cryptorchid at birth but not 3 months of age (transient cryptorchidism), and boys cryptorchid at both 0 and 3 months of age (persistent cryptorchidism) [no. (%)].

				*p*-Value		
	A: Normal boys (*n* = 2,215)^a^	B: Boys with transient cryptorchidism (*n* = 88)	C: Boys with persistent cryptorchidism (*n* = 33)	A versus B	A versus C	B versus C
Drinks/week						
0	1,539 (70.0)	43 (50.0)	23 (69.7)			
0.1–4.9	633 (28.8)	38 (44.2)	9 (27.3)	< 0.001	0.671	0.152
≥ 5	28 (1.3)	5 (5.8)	1 (3.0)			
Binge episodes						
Yes	653 (30.0)	31 (36.9)	9 (29.0)	0.178	0.905	0.432
No	1,522 (70.0)	53 (63.1)	22 (71.0)			

The *p*-value (two-sided) describes differences between the groups tested two by two.

a Two boys (normal at birth, but ascensus testis before 3 months) and 158 boys (seen at birth but not at 3 months) are not included.
